# Testosterone deficiency caused by castration increases adiposity in male rats in a tissue-specific and diet-dependent manner

**DOI:** 10.1186/s12263-020-00673-1

**Published:** 2020-08-17

**Authors:** Myunggi Baik, Jin Young Jeong, Seung Ju Park, Seon Pil Yoo, Jin Oh. Lee, Jae Sung Lee, Md Najmul Haque, Hyun-Jeong Lee

**Affiliations:** 1 Department of Agricultural Biotechnology, College of Agriculture and Life Sciences, Seoul National University, 1 Gwanak-ro, Gwanak-gu, Seoul, 08826 Republic of Korea; 2 Research Institute of Agriculture and Life Sciences, College of Agriculture and Life Sciences, Seoul National University, Seoul, Republic of Korea; 3 Institutes of Green Bio Science Technology, Pyeongchang-daero, Daehwa-myeon, Pyoengchang-gun, Gangwon-do 25354 Republic of Korea; 4 National Institute of Animal Science, Rural Development Administration, Wanju-gun, Jeollabuk-do 55365 Republic of Korea

**Keywords:** Castration, Testosterone deficiency, High-fat diet, Adiposity, Lipid and glucose metabolism

## Abstract

**Background:**

Testosterone deficiency in men is clinically associated with the development of metabolic syndrome, which manifests as obesity, hepatic steatosis, and type-2 diabetes. We investigated the effects of castration-induced testosterone deficiency on body adiposity and the expression of genes related to lipid metabolism and glucose uptake and androgen signaling in male rats fed a normal diet (ND) or a high-fat diet (HFD).

**Methods:**

Changes in lipid and glucose metabolism and androgen signaling were investigated at physiological and molecular levels in the muscle, liver, and adipose tissues of non-castrated and castrated rats under ND or HFD feeding.

**Results:**

Castration-induced testosterone deficiency predisposed animals on ND to early development of fatty liver by activating fatty acid (FA) synthesis, whereas HFD activated hepatic FA uptake *CD36* expression, leading to the development of hepatic steatosis. In rats fed ND, castration induced muscle fat accumulation by activating *CD36* expression. In the subcutaneous fat of ND-fed rats, castration increased adiposity and the expression of FA synthesis-related genes, but it decreased glucose transporter gene expression. In the abdominal fat of rats fed ND, castration increased adiposity by upregulating FA synthesis-related genes, and HFD promoted adiposity by inducing FA uptake, glucose transporter, and FA synthesis-related gene expression. In rats fed ND, castration decreased body growth and muscle weight and downregulated the expression of genes androgen signaling in the longissimus dorsi muscle.

**Conclusions:**

Testosterone deficiency increases adiposity in a tissue-specific and diet-dependent manner. Testosterone deficiency decreases body and muscle weights and downregulates androgen signaling.

## Introduction

Testosterone plays an important role in lipid and glucose metabolism [1]. Kelly et al. [2] suggested that testosterone reduces the deposition of body fat by regulating the expression of genes related to lipid and glucose metabolism. A low testosterone level or testosterone deficiency deregulates lipid and glucose metabolism, resulting in increased adiposity in the liver and peripheral tissues [2–5]. Testosterone deficiency is associated with increased visceral adiposity [6]. A low testosterone level is predictive of the development of metabolic syndrome, which manifests as obesity, nonalcoholic fatty liver disease, and type-2 diabetes in men [1, 7] and laboratory animals [2, 5]. The effects of testosterone deficiency on adiposity and lipid/glucose metabolism are controversial. For example, androgen deficiency in combination with a high-fat diet (HFD) exacerbated adiposity and insulin resistance in male mice [5]. In contrast, the serum glucose and insulin levels in male rats were unaffected by testosterone deficiency and an HFD [8]. Therefore, the effects of testosterone deficiency on lipid and glucose metabolism require further investigation.

The deposition of body fat is modulated by the lipid metabolic pathways that mediate fatty acid (FA) uptake, synthesis, and degradation [9–11]. The FA transporter CD36 mediates FA uptake and plays an important role in hepatic steatosis [12]. Limited information is available on the role of CD36 in fat deposition in testosterone-deficient rats. Kelly et al. [2] reported differences in the effect of testosterone on the function of subcutaneous (sc) versus visceral adipose tissue: testicular-feminized mice (with a non-functional androgen receptor [AR] and low testosterone level) exhibited reduced *GLUT4* expression in sc, but not visceral adipose tissue. Further studies of the effects of testosterone deficiency on the expression of genes related to lipid and glucose metabolism in peripheral tissues (including the liver and skeletal muscle) and fat depots (sc and abdominal [ab] adipose tissues) are needed to understand the mechanisms by which adiposity is regulated.

Testosterone deficiency decreases growth and muscle mass in orchidectomy and AR-knockout mice [5] and reduces muscle mass in male humans [13]. These findings suggest the importance of testosterone for body and muscle growth. Testosterone signaling is mediated by the AR signaling pathway [14, 15], and thus investigation of the effect of testosterone deficiency on AR signaling is required.

Feeding an HFD to rodents alters their lipid and glucose metabolism [16, 17] and is often used to study metabolic syndrome such as visceral adiposity, hyperlipidemia, and insulin resistance [18]. The effect of the combination of testosterone deficiency and an HFD thus needs to be clarified. We hypothesized that testosterone deficiency differentially deregulates the expression of genes related to lipid metabolism and glucose uptake in a tissue-specific manner, predisposing to metabolic syndrome. We also hypothesized that an HFD exacerbates the testosterone deficiency-induced alterations in lipid and glucose metabolism. To test these hypotheses, we examined the effect of castration on the growth, adiposity, skeletal muscle mass, blood parameters, and expression levels of genes related to lipid metabolism and glucose uptake in the liver, muscle, and sc and ab fat depots in male rats fed a normal diet (ND) or an HFD for 9 weeks.

## Materials and methods

### Animals, castration, and diets

All experimental procedures involving animals were approved by the Chonnam National University (CNU) Institutional Animal Use and Care Committee (permission number CNU IACUC-YB-R-2010-13). All animal management procedures followed the standard operating protocols of CNU. Male Sprague–Dawley rats were purchased from Orient Bio (Gyeonggi-Do, Republic of Korea) and were maintained in a temperature (22 ± 1 °C)- and humidity (45–65%)-controlled room under a 12/12 h light/dark cycle with ad libitum access to food and water*.*

Rats were castrated at 6 weeks of age, at which time their average body weight was 213 g. All surgical instruments were pre-sterilized by acceptable methods, including steam sterilization. Male rats were anesthetized by intramuscular injection of Zoletil™ 50 (5 mg/kg bodyweight, Virbac, France) and placed on a heating pad. The scrotum of the rats was shaved and cleaned using 70% ethanol. A small midline incision (1 cm) was made through the skin of the scrotum, and the testes were located, gently squeezed out, and removed. Next, the vas deferens, fat, and blood vessels were restored to their original position in the scrotal sac, and the vas deferens and blood vessels were ligated using silk sutures (Ailee, Seoul, Korea). The muscle layer and skin were closed with sutures, after which the rats were returned to their cages and their recovery monitored. The sham surgery control rats underwent an identical procedure as castrated rats except no removal of the testes.

After acclimatization for 1 week following castration with ND feeding, the rats were divided into the following four groups (8 per group): sham-operated control rats fed an ND or HFD and castrated rats fed an ND or HFD. The normal AIN93-G diet (D10012G) and HFD (D12451) were purchased from Research Diets, Inc. (New Brunswick, NJ, USA); their compositions are shown in Supporting Tables S1-S3. Briefly, in the HFD versus ND, 45% versus 16% of energy was from fat, 35% versus 64% from carbohydrates, and 20% versus 20% from protein, respectively. The food intake of the rats was recorded daily at the same time of day, and their body weight was measured weekly.

### Blood and tissue collection

At the end of the 9-week feeding period, the rats (16 weeks of age) were fasted for 8 h. The rats were anesthetized with CO_2_, and blood samples were collected via cardiac puncture, transferred into an ET tube without anticoagulant, and stored at 4 °C. Blood samples were centrifuged at 2000×*g* for 20 min at 4 °C to collect serum, which was stored at − 80 °C until analysis. After anesthetizing with CO_2_, the rats were euthanized by decapitation, and the liver, adipose tissues (ab and sc), and skeletal muscle tissues (longissimus dorsi, gastrocnemius, and soleus) were immediately removed and weighed. The tissues were frozen in liquid nitrogen and stored at − 80 °C for subsequent determination of total lipid, mRNA, and protein levels.

### Serum and tissue analyses

Serum parameters were analyzed as reported previously [19, 20]. Briefly, the serum level of glucose was analyzed using hexokinase reagents (Green Cross Reference Laboratory, Seoul, Korea). The serum level of triglycerides was analyzed by enzymatic spectrophotometric assay (Green Cross Reference Laboratory). The serum levels of testosterone and insulin were analyzed by enzyme-linked immunosorbent assay. The serum level of free FAs was analyzed by the acyl-coenzyme A (CoA) synthase–acyl-CoA oxidase method. The serum levels of total cholesterol, high-density lipoprotein (HDL), and low-density lipoprotein (LDL) were analyzed by enzymatic colorimetric assays.

Muscle tissues were dissected and placed in liquid nitrogen. Total lipids were extracted from approximately 50 mg muscle tissue by the Folch method [21].

### Histology

For histological analysis, the liver was dissected and freed from any visible fat and blood. Histologic analysis and Oil red O (ORO) staining of tissues were performed as reported previously [20]. Briefly, liver specimens were fixed in 10% formalin, equilibrated in 20% sucrose, embedded in optimal cutting temperature compound, and cut into 7-μm-thick sections using a cryostat at − 20 °C. To evaluate fat deposition, frozen sections were fixed in 10% formalin, stained with 0.5% ORO, and examined under a microscope. The degree of liver ORO staining was determined and graded from 0 to 5 (lowest to highest).

### Real-time polymerase chain reaction

Total RNA was isolated using TRIzol reagent (Molecular Research Center, Cincinnati, OH, USA) according to the manufacturer’s instructions. Total RNA was quantified by measuring the absorbance at 260 nm, and its integrity was assessed by agarose gel electrophoresis and ethidium bromide staining of the 28S and 18S RNA bands. cDNA synthesis and real-time polymerase chain reaction analyses were performed as described previously [19]. The primers were designed using Integrated DNA Technology (Coralville, IA, USA) and the Primer 3 software based on sequences in GenBank and were synthesized by Bioneer (Daejeon, Korea). Information on the primers is shown in Supporting Table S4. The 2^−ΔΔCT^ method was used to determine relative fold changes in gene expression levels normalized to the appropriate housekeeping gene (glyceraldehyde-3-phosphate dehydrogenase for liver, β-actin for muscle, and acidic ribosomal phosphoprotein P0 for adipose tissue).

### Western blotting

Western blotting was performed as described previously with slight modifications [20]. Briefly, samples were homogenized in protein extraction solution using a Polytron homogenizer (Thomas Scientific, Swedesboro, NJ, USA). The protein concentration of the samples was determined using the Bicinchoninic Acid Protein Assay Kit (Pierce, Rockford, IL, USA). Proteins were resolved by sodium dodecyl sulfate–polyacrylamide gel electrophoresis (SDS-PAGE) on either 6% (for analysis of v-Akt murine thymoma viral oncogene homolog 1 [Akt] and phospho [p]-Akt, 60 kDa) or 10% (for analysis of insulin receptor substrate-1 [IRS-1] and p-IRS-1, 180 kDa) polyacrylamide gels. The resolved proteins were transferred onto polyvinylidene fluoride membranes, blocked with 1× phosphate-buffered saline/0.1% Tween 20 containing 5% non-fat dried milk, and incubated with the appropriate primary antibodies against Akt, p-Akt, IRS-1, and p-IRS-1 (1:500 dilution). The mouse polyclonal anti-Akt, mouse monoclonal anti-p-Akt (Thr308), mouse polyclonal anti-IRS-1, and mouse monoclonal anti-p-IRS-1 antibodies were purchased from Cell Signaling Technologies (Danvers, MA, USA). Next, the blots were incubated in the presence of horseradish peroxidase-conjugated anti-mouse secondary antibodies (1:3000 dilution) (Santa Cruz Biotechnology, Santa Cruz, CA, USA) and developed using an enhanced chemiluminescence system (Thermo Scientific, Rockford, IL, USA). The processed blots were exposed to X-ray film, and the autoradiograms were analyzed using an HP scanner and Alpha Ease FC software. Band densities were normalized to that of the respective β-tubulin band.

### Statistical analyses

Data are presented as means ± standard error (SE). Data were analyzed using the general linear model procedure in SAS (SAS Institute Inc., Cary, NC, USA). The model included the effects of castration, diet (ND or HFD), and the castration × diet interaction. When interactions were significant, the PDIFF option of LSMEANS in SAS was used to perform individual comparisons of the means. Differences were considered significant when the *P* value was less than 0.05.

## Results

### Castration decreased body and muscle weight but increased body adiposity

**Fig. 1:**
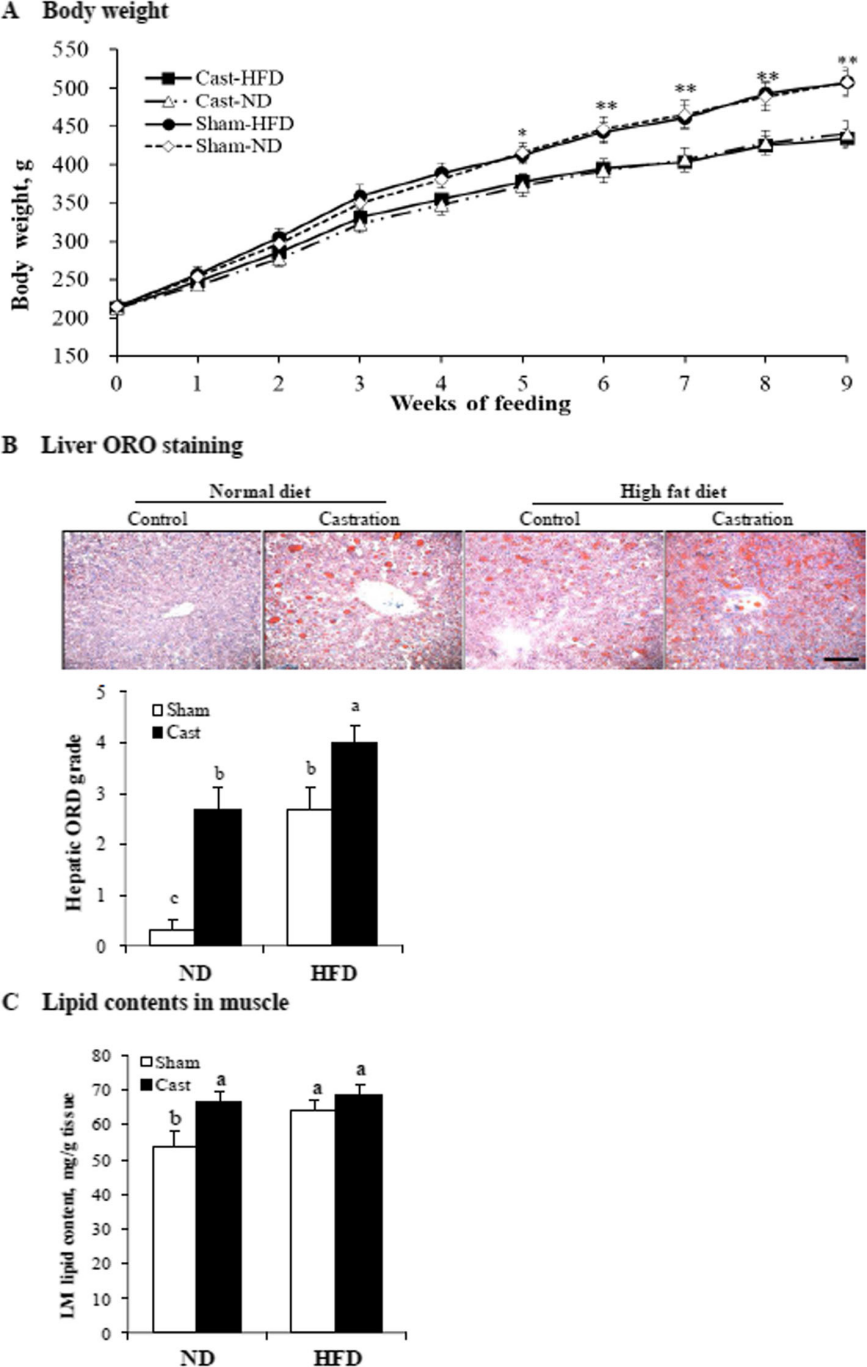
Body weight (**a**), Oil red O (ORO) staining in the liver (**b**), and lipid contents in longissimus dorsi (LD) muscle tissues (**c**) of sham-operated (Sham) and castrated (Cast) male rats fed an ND or high fat diet (HFD). **a** Values are means + SE (*n* = 7–8 rats per group). *P < 0.05 and **P < 0.01, significant differences between the Sham and Cast groups. **b** Scoring of hepatic lipid droplet accumulation (0 = none, 5 = highest). Black bar, 100 μm. **b**, **c** Values are means + SE (*n* = 7–8 per group). ^a–c^Means with different superscripts differ (*P* < 0.05)

Castrated rats in the ND and HFD groups had a lower (*P* < 0.05) body weight from 5 to 9 weeks of feeding than that of rats in the sham-control group (Fig. 1a). Castration decreased (*P* < 0.05) the food intake of the rats, and the castrated rats showed a lower (*P* < 0.05) feed efficiency than that of the control rats (Table 1).

**Table 1:** Food intake, food efficiency, and tissue weight of sham-operated (control) and castrated male rats fed either normal diet or high fat diet for 9 weeks

Items	Normal diet		High-fat diet		*P*		
	Control	Castration	Control	Castration	Castration	Diet	Interaction
Average food intake^1^, g/d	20.0 ± 0.3^b^	19.2 ± 0.3^c^	21.4 ± 0.4^a^	19.3 ± 0.4^c^	< 0.001	< 0.001	< 0.001
Initial body weight, g	254.0 ± 7.9	242.4 ± 8.7	257.3 ± 10.2	247.5 ± 8.1	NS	NS	NS
Final body weight at 9 weeks, g	507.2 ± 18.4^a^	440.9 ± 17.1^b^	507.0 ± 15.2^a^	434.4 ± 12.3^b^	< 0.001	NS	0.0019
Average food efficiency^2^, gain(g)/intake(g)	1.6 ± 0.08^a^	1.3 ± 0.07^b^	1.7 ± 0.1^a^	1.4 ± 0.09^b^	< 0.001	NS	0.0033
Abdominal fat, g	7.3 ± 0.6^c^	10.4 ± 0.7^b^	10.3 ± 0.5^b^	12.59 ± 0.6^a^	< 0.001	< 0.001	NS
Subcutaneous fat, g	18.0 ± 1.0^b^	27.2 ± 2.1^a^	21.5 ± 1.5^b^	31.2 ± 1.8^a^	< 0.001	0.04	NS
Total fat^3^, g	25.3 ± 1.4^c^	37.6 ± 2.5^b^	31.8 ± 1.6^b^	43.8 ± 2.3^a^	< 0.001	0.005	NS
Liver, g	16.2 ± 1.2^a^	14.5 ± 1.5^ab^	15.9 ± 1.1^a^	11.7 ± 0.6^b^	0.02	NS	NS
Longissimus dorsi muscle, g	10.2 ± 0.4^a^	7.9 ± 0.4^c^	9.1 ± 0.3^b^	7.2 ± 0.3^c^	< 0.001	0.01	NS
Gastrocnemius, g	5.5 ± 0.2^a^	5.0 ± 0.1^b^	5.0 ± 0.1^b^	4.6 ± 0.1^b^	< 0.001	0.001	NS
Soleus, g	0.4 ± 0.04	0.4 ± 0.06	0.5 ± 0.06	0.5 ± 0.07	NS	NS	NS

Mean ± SE (*n* = 7–8)

*NS* not significant

^1^Average food intake (g/day): average food intake for whole experimental period (9 weeks)

^2^Average food efficiency = average weight gain/food intake (g/day) for 9 weeks

^3^Total fat = subcutaneous + abdominal fat

^a–d^Means in row with different superscripts differ (*P* < 0.05)

Castration increased (*P* < 0.05) the sc, ab, and total (sc + ab) fat weights in the ND and HFD groups (Table 1). The liver weight was lower (*P* < 0.05) in the HFD group compared with the control group. The decreased liver weight may be due to decreased body weights in castrated animals fed HFD in this study. However, castration increased (*P* < 0.05) hepatic lipid accumulation in the ND group (Fig. 1b); this increase was significantly exacerbated by the HFD (*P* < 0.05). Castration reduced (*P* < 0.05) the longissimus dorsi muscle weight in the ND and HFD groups and reduced (*P* < 0.05) the gastrocnemius muscle weight in the ND group (Table 1). Castration increased (*P* < 0.05) the longissimus dorsi muscle lipid content in the ND group but not in the HFD group (Fig. 1c).

### Castration influenced the serum glucose, free FA, and cholesterol concentrations

As expected, castration decreased (*P* < 0.001) the serum concentration of testosterone irrespective of the type of diet (Table 2). Castration increased (*P* < 0.05) the serum concentration of free FAs in the ND group but not in the HFD group. Castration increased (*P* < 0.05) the serum concentrations of total cholesterol, LDL, HDL, and glucose in the ND group. Castration did not affect the serum concentration of insulin or triglycerides. HFD feeding decreased (*P* < 0.001) the serum concentration of insulin but increased (*P* < 0.05) the serum concentrations of total cholesterol and LDL. HFD may impair glucose signaling via suppressing insulin secretion in β-cell, as suggested by previous study [22].

**Table 2:** The serum parameters of sham-operated (control) and castrated-male rats fed either normal diet or high-fat diet at 8-h fasted state

Item	Normal diet		High-fat diet		*P*		
	Control	Castration	Control	Castration	Castration	Diet	Interaction
Testosterone, pg/mL	8.30 ± 1.04^a^	2.39 ± 0.13^b^	11.0 ± 1.57^a^	1.73 ± 0.39^b^	< 0.001	NS	NS
Free fatty acid, μEq/L	439 ± 43.3^b^	649 ± 41.3^a^	418 ± 31.7^b^	533 ± 25.4^b^	< 0.001	NS	NS
Insulin, ng/mL	4.27 ± 0.38^a^	4.25 ± 0.23^a^	2.43 ± 0.34^b^	1.38 ± 0.31^b^	NS	< 0.001	NS
Total cholesterol, mg/dL	78.5 ± 2.87^b^	105 ± 3.74^a^	97.5 ± 4.50^a^	112 ± 6.17^a^	< 0.001	0.02	NS
LDL cholesterol, mg/dL	7.1 ± 0.56^b^	10.2 ± 1.57^a^	15.7 ± 1.30^a^	13.2 ± 0.69^a^	NS	< 0.001	0.04
HDL cholesterol, mg/dL	69.4 ± 2.76^b^	95.1 ± 4.59^a^	83.3 ± 5.03^a^	95.7 ± 5.00^a^	< 0.001	NS	NS
Triglyceride mg/dL	100 ± 12.3	93.7 ± 8.30	81.7 ± 8.35	78.1 ± 7.24	NS	NS	NS
Glucose, mg/dL	179 ± 17.1^b^	237 ± 10.9^a^	190 ± 16.2^b^	215 ± 10.1^ab^	0.01	NS	NS

Mean ± SE (*n* = 7–8)

*NS* not significant

^a-b^Means in row with different superscripts differ (*P* < 0.05)

### Castration and an HFD modulate the expression of genes related to lipid metabolism and glucose uptake

**Fig. 2:**
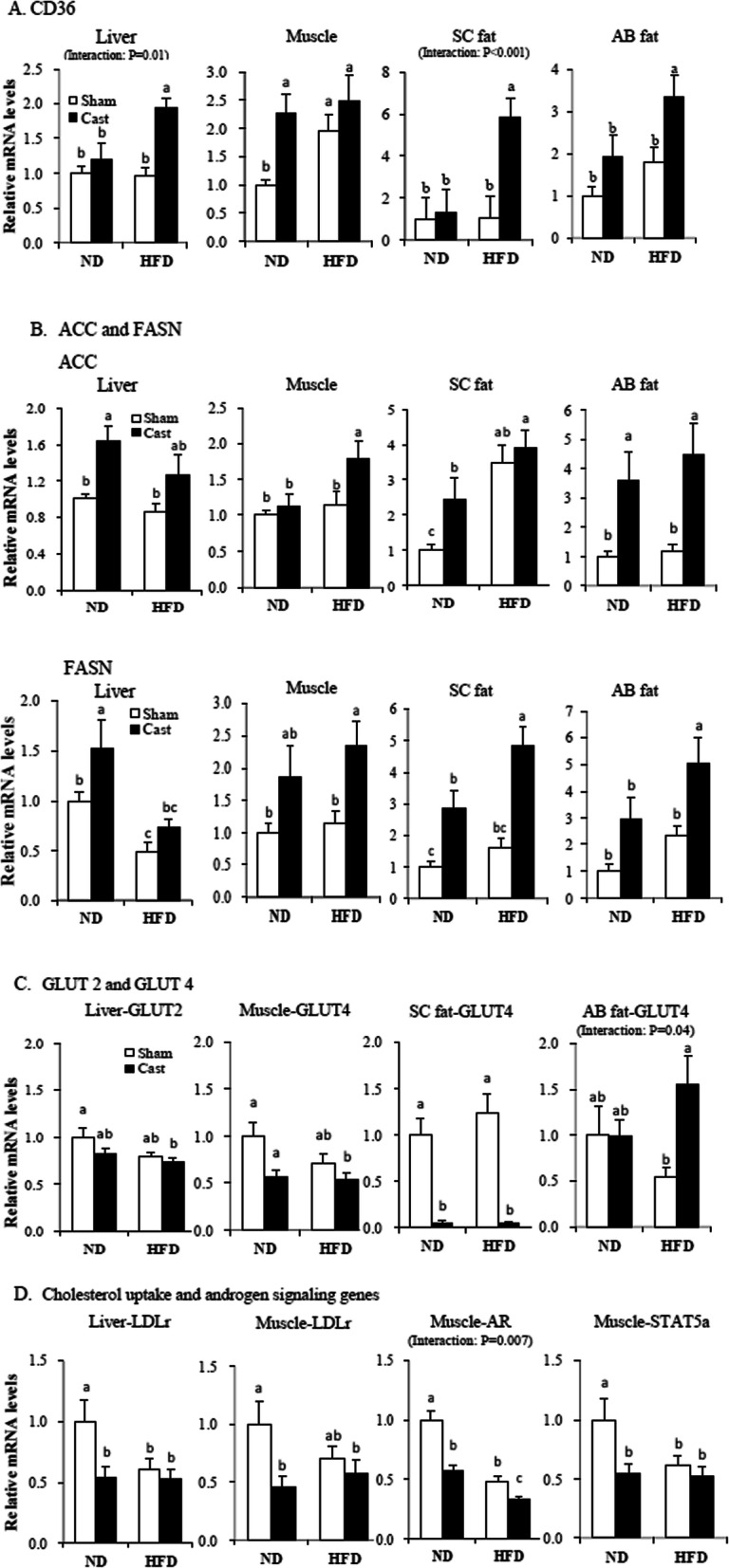
Expression levels of genes related to FA uptake (**a**), FA synthesis (**b**), glucose transporter (**c**), and cholesterol uptake and androgen signaling (**d**) in the liver, muscle, subcutaneous fat (sc), and abdominal fat (ab) of sham-operated (Sham) and castrated (Cast) male rats fed a normal diet (ND) or high fat diet (HFD). mRNA levels were determined by real-time PCR, and the values of sham-operated rats fed the ND were normalized to 1.0. Values are means + SE (*n* = 7–8). ^a–c^Means with different superscripts differ (*P* < 0.05). *CD36* cluster of differentiation 36, *ACC* acetyl-coA carboxylase, *FASN* fatty acid synthase, *GLUT2* glucose transporter 2, *GLUT4* glucose transporter 4, *LDLr* low-density lipoprotein receptor, *AR* androgen receptor, *STAT5a* signal transducer and activator of transcription 5a

Castration modulated the expression of genes related to lipid metabolism in a tissue-specific manner. Castration upregulated (*P* < 0.05) the expression of *CD36* in the liver and in sc and ab fat in the HFD group but not in the ND group, while it upregulated (*P* < 0.05) the expression of *CD36* in the muscle in the ND group but not in the HFD group (Fig. 2a). In the liver, castration upregulated (*P* < 0.05) the expression of acetyl-coA carboxylase (*ACC*) and fatty acid synthase (*FASN*) in the ND group. In contrast, castration upregulated (*P* < 0.05) the expression of *ACC* and *FASN* in muscle in the HFD group (Fig. 2b). In sc and ab fat, castration upregulated (*P* < 0.05) the expression of *ACC* and/or *FASN* in both groups.

Castration did not affect *GLUT2 expression* in the liver or *GLUT4* expression in muscle in both diet groups (Fig. 2c). In contrast, castration downregulated (*P* < 0.05) the expression of *GLUT4* in sc fat in both groups (3–10% of the level in the control group). In ab fat, castration upregulated *GLUT4* expression in the HFD group but not in the ND group. The HFD decreased (*P* < 0.05) *glucose transporter* expression in the liver but not in the other tissues evaluated. Castration decreased (*P* < 0.05) the expression of LDL receptor (*LDLr*) in the liver and muscle in the ND group (Fig. 2d). In muscle tissues, castration decreased *AR* expression in both groups (Fig. 2d) and signal transducer and activator of transcription 5a (*STAT5a*) expression in the ND group. The HFD decreased (*P* < 0.05) the expression of *AR* in the muscle. Neither castration nor diet type affected *STAT5a* and *AR* expression in either the liver or sc fat (data not shown). Neither castration nor diet type affected the protein level of IRS-1, p-IRS-1, Akt, or p-Akt in any of the muscle tissues (Supporting Fig. S1).

## Discussion

### Testosterone deficiency increases body adiposity and deregulates expression of genes related to lipid metabolism and glucose uptake in tissue-specific and diet-dependent manners

In this study, we found that castration-induced testosterone deficiency increased adiposity in the liver, muscle, and sc and ab fat in rats fed an ND and also increased adiposity in the liver and sc and ab fat, but not in the muscle, in rats fed an HFD. Consistent with our results, a low testosterone level or testosterone deficiency reportedly increases adiposity in the liver and several peripheral tissues [2, 3, 5], and an HFD increases tissue adiposity [4, 5, 18].

We examined the expression levels of genes involved in FA uptake and synthesis to evaluate the molecular mechanisms underlying the effects of testosterone deficiency and diet type on adiposity. Castration increased the expression of *CD36* in the liver and sc and ab fat in the HFD group, but not in the ND group, and in the muscle in the ND group, but not in the HFD group. The activation of CD36 is involved in diet-induced hepatic steatosis [23, 24] and is linked to non-alcoholic fatty liver disease in humans [25]. However, activation of *CD36* expression in adipose fat cells is unclear. Our findings indicate that HFD-induced upregulation of *CD36* expression is associated with increased sc and ab adiposity in addition to hepatic steatosis. Castration increased the expression of *ACC* and *FASN* in the liver in the ND group but in muscle in the HFD group. In sc and ab fat, castration increased the expression of *ACC* and/or *FASN* in both diet groups; the increased *FASN* expression in sc fat is consistent with a previous study involving mice [5].

Castration also modulated the expression of genes related to glucose uptake. In sc fat, castration markedly decreased the expression of *GLUT4* in the ND and HFD groups. In contrast, castration did not affect the expression of genes related to glucose uptake in the liver or muscle in the ND or HFD group. In ab fat, castration increased the expression of *GLUT4* in the HFD, but not in the ND, group. Similarly, Kelley et al. [2] reported that *GLUT4* expression was decreased in sc fat but not in ab fat in testicular-feminized compared with normal mice. In this study, the circulating glucose concentration was increased in castrated rats fed an ND but not in those fed an HFD. The increased glucose concentrations may be due to combined effects of the decreased GLUT4 gene expression in sc fat and the decreased muscle mass observed in castrated animals.

Collectively, our results suggest that testosterone deficiency regulates adiposity in a tissue-specific and diet-dependent manner (Supporting Fig. S2). Upregulation of *ACC* and *FASN* expression may have contributed to the initial increase in hepatic adiposity in rats fed an ND, and activation of *CD36* expression was responsible for the development of hepatic steatosis in rats fed an HFD. The increased adiposity in the muscle tissues of rats in the ND group may be due to FA uptake because the expression of *CD36*, but not that of *ACC*, *FASN*, or *GLUT4*, was increased. In sc fat, the expression of genes related to FA synthesis was upregulated and that of genes related to glucose transporter was downregulated in the ND and HFD groups. Glucose is a major substrate for FA synthesis in fat cells [26]. Thus, further clarification of the contribution of FA synthesis to the castration-induced increase in sc fat accumulation is needed. In ab fat, upregulation of genes related to FA synthesis may have contributed to the ND-induced increase in adiposity. Moreover, an HFD may aggravate ab adiposity by activating the expression of genes related to FA uptake (*CD36*) and synthesis, as *GLUT4* expression was increased in the HFD group but unaffected in the ND group. In other words, in ab fat, a portion of glucose precursors for FA synthesis may be derived from the castration-induced increase in circulating glucose concentration. Overall, our results suggest that a normal circulating testosterone level is important for regulating FA uptake and synthesis in all tissues and that testosterone deficiency differentially deregulates lipid metabolism. This may result in elevation of the circulating free FA and cholesterol concentrations, leading to adiposity in the liver, muscle, and ab and sc fat depots.

Our results also suggest that a low testosterone level is associated with an increased risk of type-2 diabetes, in that castrated rats exhibited reduced expression of genes related to glucose uptake in sc fat as well as an elevated circulating glucose concentration. Following castration, we found increased blood cholesterol levels, which may be caused by decreased cholesterol uptake in liver and muscle, as the expression of *LDLr* in the liver and muscle was decreased by castration of the rats.

The increased adiposity of the liver and fat tissues in the castrated rats may have been differentially induced in the ND and HFD groups. The ND contained more carbohydrate (64%) and less fat (7%) than the HFD (41% carbohydrate and 24% fat). The higher insulin concentrations observed in rats fed an ND, compared with those fed an HFD, may reflect higher carbohydrate consumption. In the ND group, the castrated rats had higher glucose concentrations than non-castrated animals; the elevated glucose concentrations in the castrated rats may partially contribute to the increased adiposity in liver and fat tissues. In the ND group, the increased *ACC* and *FASN* expression levels in the liver and sc and ab fat of the castrated rats, compared with non-castrated rats, support our assumption that high carbohydrate consumption causes elevated adiposity in these tissues, although there is a need to consider the reduced *GLUT4* expression in the sc fat of the castrated rats, compared with non-castrated rats. Meanwhile, the rats fed an HFD had higher total cholesterol concentrations than rats fed an ND. The elevated total cholesterol concentrations in the castrated rats fed an HFD may partially contribute to the increased adiposity in the liver and in ab and sc fats. In the HFD group, the increased CD36 expression in the liver and ab and sc fat of the castrated rats, compared with non-castrated rats, may partially support the implication that high fat consumption causes the increased adiposity in these tissues.

### Testosterone deficiency decreases body and muscle weights and downregulates androgen signaling

We found that castration decreased the body, longissimus dorsi, and gastrocnemius weights of the rats. Castration also decreased *STAT5a* and *AR* expression in the longissimus dorsi muscle, and *AR* expression was further decreased by the HFD. Thus, castration-induced testosterone deficiency deregulates AR signaling, and a normal testosterone level is required for AR signaling. Furthermore, the HFD augmented the castration-induced decrease in muscle mass and *AR* expression. Therefore, proper nutrition is important for maintaining muscle mass in the presence of a low testosterone level because a low testosterone level may aggravate the HFD-induced decrease in muscle mass. Decreased muscle mass may decrease glucose utilization by muscles, leading to hyperglycemia and deregulation of glucose homeostasis. This may in part explain the increased ab adiposity under testosterone deficiency in the ND and HFD groups.

Zirkin and Tenover [27] reported that the circulating testosterone level typically declines with age. Indeed, treatment of older men with growth hormone and testosterone improved their lean body mass [28]. Testosterone deficiency, which can be caused by hypogonadism, central obesity, or androgen-deprivation in prostate cancer patients, is related with metabolic syndromes including insulin resistance and type-2 diabetes [29]. In animals, an HFD differentially influences body metabolism and metabolic disease with age. HFD feeding resulted in greater increases in body weight and serum total cholesterol and glucose levels in older mice compared with younger mice [30]. Our study suggests that consumption of an HFD by elderly men, who generally have low testosterone levels, may promote the development of metabolic syndrome.

In this study, the rats were castrated at 6 weeks of age, when they were immature. Consequently, castration may have affected the overall development of the animals and contributed to physiological differences relative to non-castrated animals. In men, testosterone levels typically decline at old age. Therefore, there may be a limitation for direct implication of our findings to men, since rats were castrated at immature age.

A testosterone supplementation study to the castrated rats could validate the testosterone deficiency model by castration, although we did not conduct the experiment.

## Conclusions

In conclusion, castration-induced testosterone deficiency influenced body adiposity and the expression of genes related to lipid metabolism and glucose uptake in a tissue-specific and diet-dependent manner in rats (Supporting Fig. S2). Testosterone deficiency initially induced hepatic fat accumulation by activating the expression of genes related to FA synthesis; the HFD increased hepatic fat accumulation further by upregulating genes related to FA uptake. In muscle, testosterone deficiency in rats fed the ND, but not in those fed the HFD, induced fat accumulation by activating *CD36* expression. In sc fat, contribution of glucose for FA synthesis is questionable for the castration-induced increase in adiposity because castration decreased glucose transporter expression in both diet groups although it increased FA synthesis gene expression. Upregulation of genes related to FA uptake may be involved in the HFD-induced increase in sc fat. The ab fat accumulation of castrated rats fed a ND may be increased via upregulation of genes related to FA synthesis and that fed an HFD was further induced via upregulation of genes related to FA uptake, glucose transporter, and FA synthesis. The castration-induced downregulation of genes related to glucose transporter in sc fat and increase in the circulating glucose concentration imply the involvement of testosterone deficiency in the pathogenesis of type-2 diabetes.

## Supplementary information

**Additional file 1: Table S1.** Composition of the experimental diets (g/kg). **Table S2.** Composition of mineral mix in experimental diets. **Table S3.** Composition of vitamin mix in experimental diets. **Table 4.** Primers sequences used in real-time PCR. **Figure S1.** Summary of castration effects on adiposity (TG accumulation), blood parameters, and gene expression under normal diet (ND: A) and high-fat diet (HFD) feeding (B) in liver, longissimus dorsi (LD) muscle, and subcutaneous (sc) and abdominal (ab) fats of male rats. **Figure S2.** Summary of castration effects on adiposity (TG accumulation), blood parameters, and gene expression under normal diet (ND: A) and high-fat diet (HFD) feeding (B) in liver, *longissimus dorsi* (LD) muscle, and subcutaneous (sc) and abdominal (ab) fats of male rats. The arrow indicates changes (up or down) of parameters by castration. *T* testosterone, *TG* triglycerol, *FFA* free fatty acid, *CD36* cluster of differentiation 36, *ACC* acetyl-CoA carboxylase, *FASN* fatty acid synthase, *GLUT2* glucose transporter 2, *GLUT4* glucose transporter 4, *LDLr* low density lipoprotein receptor, *AR* androgen receptor, *STAT5a* signal transducer and activator of transcription 5a. ND = no difference.

## Data Availability

Not applicable

## Abbreviations

ACC: Acetyl-CoA carboxylase
AR: Androgen receptor
CD36: Fatty acid translocase
FA: Fatty acid
FASN: Fatty acid synthase
GLUT2: Glucose transporter 2
GLUT4: Glucose transporter 4
HFD: High-fat diet
LDLr: Low-density lipoprotein receptor
ND: Normal diet
