# Supplementary material for: Testosterone deficiency caused by castration increases adiposity in male rats in a tissue-specific and diet-dependent manner
Source: Genes Nutr. 2020 Aug 17;15:14. doi: 10.1186/s12263-020-00673-1 (PMC7433145; doi:10.1186/s12263-020-00673-1)
Supplement: Supplementary file 1 — Additional file 1: Table S1. Composition of the experimental diets (g/kg). Table S2. Composition of mineral mix in experimental diets. Table S3. Composition of vitamin mix in experimental diets. Table 4. Primers sequences used in real-time PCR. Figure S1. Summary of castration effects on adiposity (TG accumulation), blood parameters, and gene expression under normal diet (ND: A) and high-fat diet (HFD) feeding (B) in liver, longissimus dorsi (LD) muscle, and subcutaneous (sc) and abdominal (ab) fats of male rats. Figure S2. Summary of castration effects on adiposity (TG accumulation), blood parameters, and gene expression under normal diet (ND: A) and high-fat diet (HFD) feeding (B) in liver, longissimus dorsi (LD) muscle, and subcutaneous (sc) and abdominal (ab) fats of male rats. The arrow indicates changes (up or down) of parameters by castration. T testosterone, TG triglycerol, FFA free fatty acid, CD36 cluster of differentiation 36, ACC acetyl-CoA carboxylase, FASN fatty acid synthase, GLUT2 glucose transporter 2, GLUT4 glucose transporter 4, LDLr low density lipoprotein receptor, AR androgen receptor, STAT5a signal transducer and activator of transcription 5a. ND = no difference. [file 12263_2020_673_MOESM1_ESM.doc]

**Supplementary information**

**Table S1.** Composition of the experimental diets (g/kg)

| Ingredient | AIN-93G | |  | High fat diet (HFD)-45% | |
| --- | --- | --- | --- | --- | --- |
|  | g/kg | kcal/kg |  | g/kg | kcal/kg |
| Casein | 200 | 800 |  | 200 | 800 |
| L-Cystine | 3 | 12 |  | 3 | 12 |
| Corn Starch | 397 | 1590 |  | 72.8 | 291 |
| Maltodextrin 10 | 132 | 528 |  | 100 | 400 |
| Sucrose | 100 | 400 |  | 176.8 | 691 |
| Cellulose | 50 | 0 |  | 50 | 0 |
| Soybean Oil | 70 | 630 |  | 25 | 225 |
| t-Butylhydroquinone | 0.014 | 0 |  | - | - |
| Lard | - | - |  | 177.5 | 1598 |
| Mineral Mix | 35 S10022G | 0 S10022G |  | 10 S10026 | 0 S10026 |
| DiCalcium Phosphate | - | - |  | 13 | 0 |
| Calcium Carbonate | - | - |  | 5.5 | 0 |
| Potassium Citrate , H_2_O | - | - |  | 16.5 | 0 |
| Vitamin Mix^1^ | 10 V10037 | 40 V10037 |  | 10 V10001 | 40 V10001 |
| Choline Bitartrate | 2.5 | 0 |  | 2 | 0 |
| FD&C Blue Dye#1 | - | - |  | 0.05 | 0 |
| Total energy, kJ/g |  | 16.7 |  |  | 17.0 |
| Composition | Gram% | Kcal% |  | Gram% | Kcal% |
| Protein | 20.0 | 20.3 |  | 24.0 | 20.0 |
| Carbohydrate | 64.0 | 63.9 |  | 41.0 | 35.0 |
| Fat | 7.0 | 15.8 |  | 24.0 | 45.0 |

4,000kcal/kg = 16.7MJ/kg; 4,057kcal/kg = 17.0MJ/kg

This study used D10012G and D12451 diets (Research Diets) as AIN-93G and HFD 45%, respectively. Composition of diets was obtained from the Research Diets Web site (http://www.researchdiets.com/).

^1^Formula of mineral and vitamin mixes is shown in supporting Tables 2 and 3, respectively.

**Table S2. Composition of mineral mix in experimental diets**

| Ingredient |  | |  | Mineral Mix S10026 for high-fat diet | | Mineral Mix S10022G for AIN93G |
| --- | --- | --- | --- | --- | --- | --- |
|  |  | |  | g |  | g |
| Sodium chloride | | |  | 259 |  | 74 |
| Magnesium oxide | | |  | 41.9 |  | 24 |
| Magnesium sulfate | | |  | 257.6 |  | - |
| Ammonium molybdate | | |  | 0.3 |  | 0.00795 |
| Chromium potassium sulfate | | | | 1.925 |  | 0.275 |
| Copper carbonate | | |  | 1.05 |  | - |
| Ferric citrate | | |  | 21 |  | 6.06 |
| Manganese carbonate | | |  | 12.25 |  | 0.63 |
| Potassium iodate | | |  | 0.035 |  | 0.01 |
| Sodium fluoride | | |  | 0.2 |  | 0.0635 |
| Sodium selenite | | |  | 0.035 |  | - |
| Zinc carbonate | | |  | 5.6 |  | 1.65 |
| Calcium carbonate | | |  | - |  | 357 |
| Potassium phosphate | | |  | - |  | 196 |
| Potassium citrate | | |  | - |  | 70.78 |
| Potassium sulfate | | |  | - |  | 46.6 |
| Cupric carbonate | | |  | - |  | 0.3 |
| Sodium selenate | | |  | - |  | 0.01025 |
| Sodium silicate | | |  | - |  | 1.45 |
| Lithium chloride | | |  | - |  | 0.0174 |
| Boric acid | |  |  | - |  | 0.0815 |
| Nickel carbonate | | |  | - |  | 0.0318 |
| Ammonium vanadate | | |  | - |  | 0.0066 |
| Sucrose |  | |  | 399.105 |  | 221.026 |
| Total |  | |  | 1000 |  | 1000 |

**Table S3.** Composition of vitamin mix in experimental diets

| Ingredient |  |  | Vitamin Mix V10001 for high-fat diet | | Vitamin Mix V10037 for AIN93G |
| --- | --- | --- | --- | --- | --- |
|  |  |  | g |  | g |
| Vitamin A palmitate | |  | 0.8 |  | - |
| Vitamin A acetate | |  | - |  | 0.8 |
| Vitamin D3 | |  | 1 |  | 1 |
| Vitamin E acetate | |  | 10 |  | 15 |
| Menadione sodium bisulfite | | | 0.08 |  | - |
| Biotin, 1.0% | |  | 2 |  | 2 |
| Cyanocobalamin | |  | 1 |  | 2.5 |
| Folic acid |  |  | 0.2 |  | 0.2 |
| Nicotinic acid | |  | 3 |  | 3 |
| Calcium pantothenate | |  | 1.6 |  | 1.6 |
| Pyridoxine HCl | |  | 0.7 |  | 0.7 |
| Riboflavin |  |  | 0.6 |  | 0.6 |
| Thiamin HCl | |  | 0.6 |  | 0.6 |
| Phylloquinone | |  | - |  | 0.075 |
| Sucrose |  |  | 978.42 |  | 971.925 |
| Total |  |  | 1000 |  | 1000 |

**Table S4.** Primers sequences used in real-time PCR

| Gene name, Symbol | Accession number | Primer | Sequence | Length (bp) |
| --- | --- | --- | --- | --- |

| Fatty acid translocase/CD36, CD36 | AF072411 | Forward | ccagaacccagacaaccact | 106 |
| --- | --- | --- | --- | --- |
|  |  | Reverse | cacaggctttccttctttgc |  |
| Acetyl-CoA carboxylase , ACC | NM_022193 | Forward | aagaggtttcaggcacagtc | 142 |
|  |  | Reverse | tcagtatgtcggaaggcaaag |  |
| Fatty acid synthase, FASN | NM_017332 | Forward | ggatgtcaacaagcccaagt | 98 |
|  |  | Reverse | cagaggagaaggccacaaag |  |
| Glucose transporter 2, GLUT2 | NM_012879 | Forward | aaagccccagatacctttacc | 79 |
|  |  | Reverse | tcagtgccccttagtcttttc |  |
| Glucose transporter 4, GLUT4 | NM_012751 | Forward | agcagctctcaggcatcaatg | 70 |
|  |  | Reverse | tgttccaccccagctaactc |  |
| Androgen receptor , AR | NM_012502 | Forward | acaacaaccagcctgattcc | 133 |
|  |  | Reverse | atctggtcatccacatgcaag |  |
| Low density lipoprotein receptor, LDLr | NM_175762 | Forward | gccattttcagtgccaacc | 124 |
|  |  | Reverse | cacaccagtttacccctctag |  |
| Signal transducer and activator of transcription 5a | NM_017064 | Forward | cggctggaactataccttctg | 110 |
| STAT5a |  | Reverse | gcttgttcacgaaacccaag |  |
| Acidic ribosomal phosphoprotein P0, ARPP P0^1^ | BC062028 | Forward | gtcacagtacctgctcagaac | 139 |
|  |  | Reverse | ccttgtctccagtctttatc |  |
| Glyceraldehyde-3-phosphate dehydrogenase, | NM_017008 | Forward | tgccactcagaagactgtgg | 85 |
| GAPDH^1^ |  | Reverse | ggatgcagggatgatgttct |  |
| β-actin, ACTB^1^ | NM_031144 | Forward | gctacagcttcaccaccaca | 73 |
|  |  | Reverse | agggcaacatagcacagctt |  |

^1^Housekeeping genes: β-actin for muscle, GAPDH for liver, and ARPP P0 for adipose tissue.

A. IRS-1 and pIRS-1 protein levels

180kDa

**ND**

**HFD**


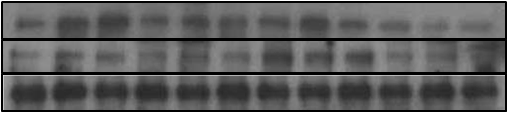


Cast

Sham

Cast

Sham

180kDa

55kDa

IRS-1

β-tubulin

pIRS-1

B. Akt and pAkt protein levels


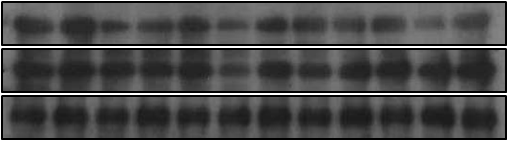


**ND**

Sham

Sham

Cast

**HFD**

Cast

60kDa

60kDa

pAkt

Akt

55kDa

β-tubulin

**Figure S1.** Protein levels of insulin signaling IRS-1 and pIRS-1 (A) and Akt and pAkt (B) in *longissimus dorsi* muscle tissues of sham-operated (Sham) and castrated (Cast)-male rats fed either normal diet (ND) or high fat diet (HFD). Protein levels were measured by Western blot. Bar graphs show quantification of protein levels. Protein levels were normalized with a housekeeping β-tubulin gene. Values of sham-operated rats fed ND were normalized to 1.0. Data are expressed as means + SEM (n=3). *Akt* v-akt murine thymoma viral oncogene homolog 1, *pAkt* phospho-Akt, *IRS-1* insulin receptor substrate 1, *pIRS-1* phospho-insulin receptor substrate 1.

1. ND feeding

**
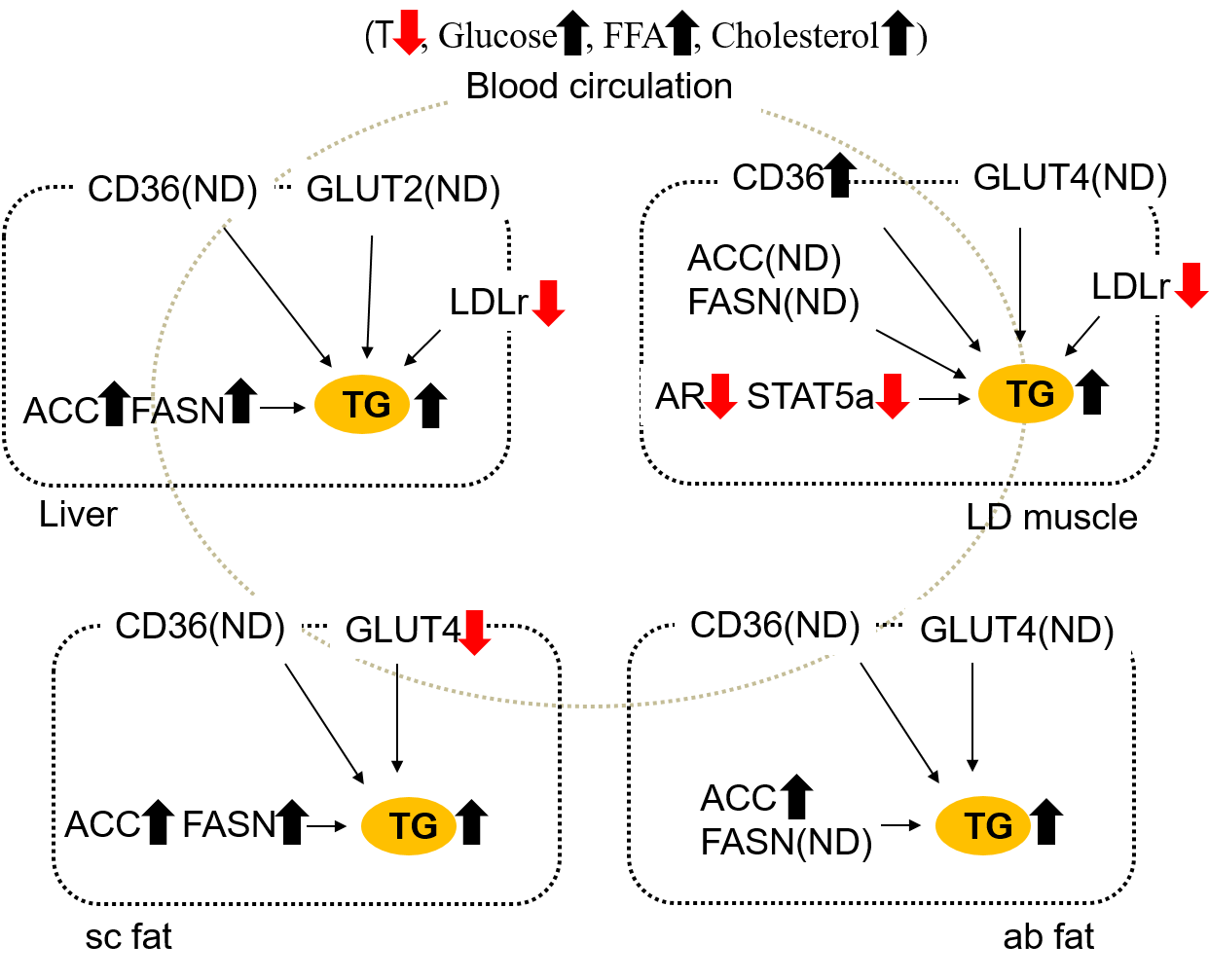
**

1. HFD feeding


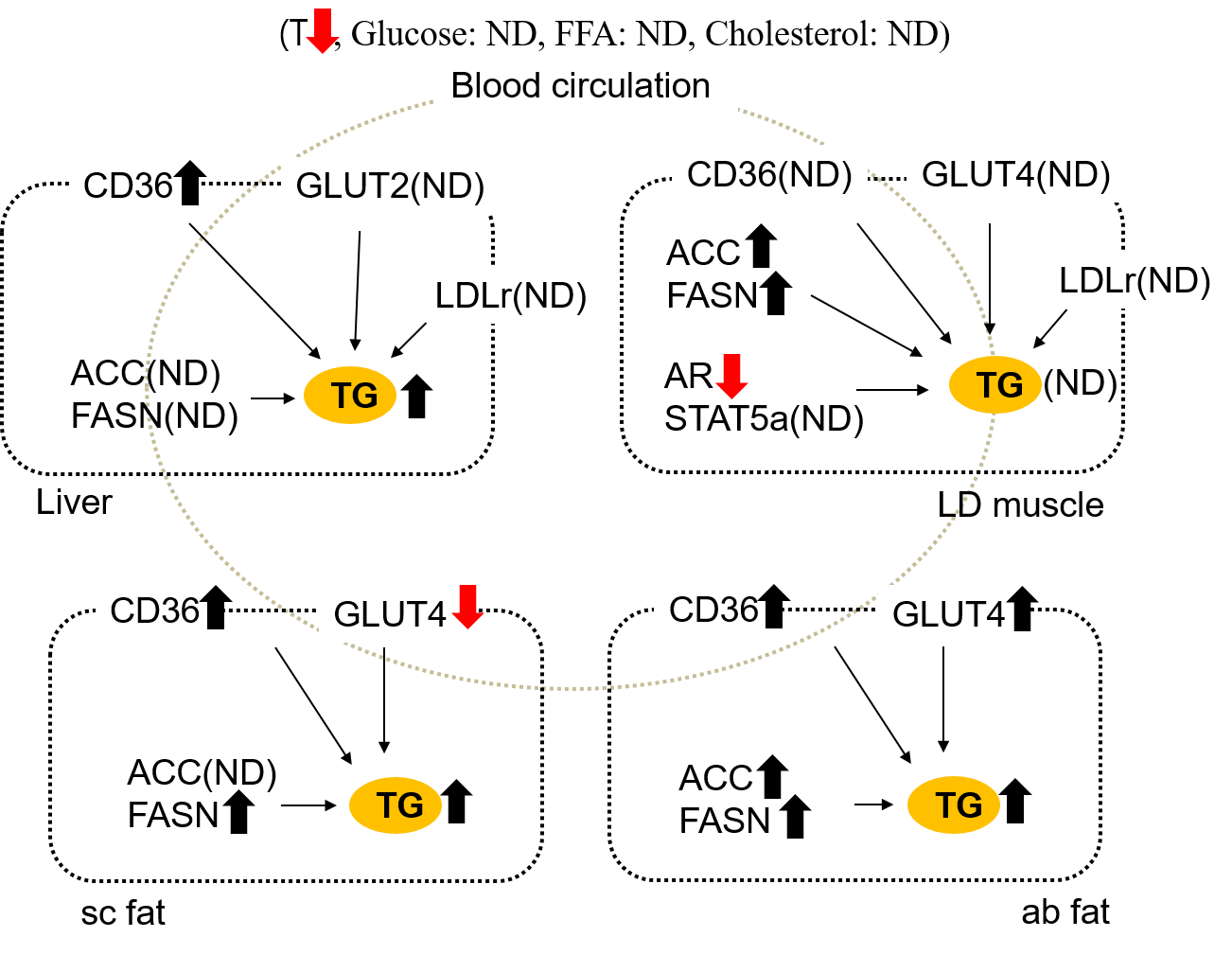


**Figure S2.** Summary of castration effects on adiposity (TG accumulation), blood parameters, and gene expression under normal diet (ND: A) and high-fat diet (HFD) feeding (B) in liver, *longissimus dorsi* (LD) muscle, and subcutaneous (sc) and abdominal (ab) fats of male rats. The arrow indicates changes (up or down) of parameters by castration. *T* testosterone, *TG* triglycerol, *FFA* free fatty acid, *CD36* cluster of differentiation 36, *ACC* acetyl-CoA carboxylase, *FASN* fatty acid synthase, *GLUT2* glucose transporter 2, *GLUT4* glucose transporter 4, *LDLr* low density lipoprotein receptor, *AR* androgen receptor, *STAT5a* signal transducer and activator of transcription 5a. ND = no difference.
